# Characterizing White Matter in Huntington's Disease

**DOI:** 10.1002/mdc3.12866

**Published:** 2019-11-28

**Authors:** Sarah Gregory, Eileanoir Johnson, Lauren M. Byrne, Filipe B. Rodrigues, Alexandra Henderson, John Moss, David Thomas, Hui Zhang, Enrico De Vita, Sarah J. Tabrizi, Geraint Rees, Rachael I. Scahill, Edward J. Wild

**Affiliations:** ^1^ University College London Huntington's Disease Centre, Department of Neurodegenerative Disease University College London Queen Square Institute of Neurology, University College London London United Kingdom; ^2^ Department of Brain Repair and Rehabilitation, University College London Queen Square Institute of Neurology University College London United Kingdom; ^3^ Leonard Wolfson Experimental Neurology Centre, University College London Queen Square Institute of Neurology University College London United Kingdom; ^4^ Department of Computer Science and Centre for Medical Image Computing University College London London United Kingdom; ^5^ Lysholm Department of Neuroradiology National Hospital for Neurology and Neurosurgery London United Kingdom; ^6^ Department of Biomedical Engineering, School of Biomedical Engineering and Imaging Sciences King's College London London United Kingdom; ^7^ Wellcome Trust Centre for Neuroimaging, Institute of Neurology University College London London United Kingdom

**Keywords:** Huntington's disease, MRI, white matter, neurofilament light (NfL)

## Abstract

**Background:**

Investigating early white matter (WM) change in Huntington's disease (HD) can improve our understanding of the way in which disease spreads from the striatum.

**Objectives:**

We provide a detailed characterization of pathology‐related WM change in HD. We first examined WM microstructure using diffusion‐weighted imaging and then investigated both underlying biological properties of WM and products of WM damage including iron, myelin plus neurofilament light, a biofluid marker of axonal degeneration—in parallel with the mutant huntingtin protein.

**Methods:**

We examined WM change in HD gene carriers from the HD–CSFcohort, baseline visit. We used standard‐diffusion magnetic resonance imaging to measure metrics including fractional anisotropy, a marker of WM integrity, and diffusivity; a novel diffusion model (neurite orientation dispersion and density imaging) to measure axonal density and organization; T1‐weighted and T2‐weighted structural magnetic resonance imaging images to derive proxy iron content and myelin‐contrast measures; and biofluid concentrations of neurofilament light (in cerebrospinal fluid (CSF) and plasma) and mutant huntingtin protein (in CSF).

**Results:**

HD gene carriers displayed reduced fractional anisotropy and increased diffusivity when compared with controls, both of which were also associated with disease progression, CSF, and mutant huntingtin protein levels. HD gene carriers also displayed proxy measures of reduced myelin contrast and iron in the striatum.

**Conclusion:**

Collectively, these findings present a more complete characterization of HD‐related microstructural brain changes. The correlation between reduced fractional anisotropy, increased axonal orientation, and biofluid markers suggest that axonal breakdown is associated with increased WM degeneration, whereas higher quantitative T2 signal and lower myelin‐contrast may indicate a process of demyelination limited to the striatum.

The ongoing development of novel therapeutics to treat Huntington's disease (HD) necessitates improved characterization of HD‐related brain changes. HD is a progressive neurodegenerative disorder characterized by a motor, cognitive, and neuropsychiatric phenotype and caused by CAG expansions in the huntingtin gene (*HTT*). Given the certainty of onset in those that inherit the gene combined with genetic testing, we can examine brain changes from the earliest, presymptomatic disease stages.

Magnetic resonance imaging (MRI) measures of neuronal atrophy have characterized macrostructural brain changes associated with HD progression.^1^, ^2^ Neurodegeneration primarily originates in the striatum, extending to white matter (WM) and finally the cortex.^1^, ^3^ However, although a robust marker of disease progression, macrostructural changes provide limited descriptions of pathological mechanisms underlying HD. Investigating early WM microstructural changes in HD can improve our understanding of WM organization and the way in which the disease spreads from deep gray matter (GM). As such, studies using diffusion tensor imaging (DTI) have shown WM disorganization during the earliest stages of premanifest HD (preHD), with increased diffusivity perpendicular (radial diffusivity [RD]) and parallel (axial diffusivity [AD]) to the main fibers.^4^, ^5^

However, the nonspecific nature of standard diffusion analysis techniques has limited our ability to identify underlying features of reduced WM organization in HD.^6^ New diffusion modeling approaches can offer improved characterization of WM properties. Neurite orientation dispersion and density imaging (NODDI), for example, uses a 3‐tissue compartment model to isolate intraneurite and extraneurite (axonal) diffusion, allowing characterization of axonal density and orientation^7^ that is congruent with histological findings.^8^ When used in the preHD TrackOn‐HD cohort,^9^ NODDI showed widespread reductions in axonal density and axonal reorganization in basal ganglia WM.^5^ More important, the technique was more sensitive to HD‐related WM change than standard diffusion imaging measures.

The investigation of underlying biological properties and products of WM damage can potentially complement diffusion imaging of HD‐related WM changes. Myelin is a crucial component for axonal structure and efficient WM functionality. Early demyelination in HD may increase iron‐rich^4^ oligodendrocytes necessary for myelin repair, leading to increased measurable iron and reduced myelin contrast.^10^ Alternatively, increased iron, without congruent decreases in myelin contrast, may simply indicate increased toxicity and neuronal loss.^11^ Both iron and myelin can be measured using MRI, and previous work in HD has shown increased basal ganglia iron levels,^12^, ^13^ with suggested widespread demyelination.^14^

Similarly, neurofilament light (NfL)—a key component of the neuronal cytoskeleton and marker of axonal degeneration—is elevated in HD gene carriers,^15^ predicting both brain atrophy and clinical and cognitive decline.^16^ *mHTT* (mutant *HTT*), the pathogenic agent in HD, and its concentration may be interpreted as a direct measure of the genetic–pathological burden in HD that appears to be neuronal in origin.^17^ These 2 proteins can be reliably quantified in cerebrospinal fluid (CSF)^18^, ^19^ and are among the earliest detectable changes in HD.^20^

Here we have attempted to characterize more fully white matter in HD by investigating the relationships between microstructural imaging markers, measures of myelin and iron content, biofluid markers (NfL and *mHTT*), and measures of disease progression in HD gene carriers from the HD‐CSF cohort.^20^ We used standard diffusion measures: fractional anisotropy (FA), mean diffusivity (MD), AD, and RD plus NODDI measures of axonal density and orientation to index WM microstructural change in addition to myelin contrast and quantitative T2 mapping as proxy measures of myelination and iron content, respectively.

## Methods

### HD‐CSF Cohort

The HD‐CSF cohort consists of 20 healthy controls, 20 preHD gene carriers, and 40 manifest HD gene carriers.^20^ Manifest HD gene carriers had a CAG repeat length ≥36 and a Unified Huntington's Disease Rating Scale diagnostic confidence score = 4, with HD stage defined according to the Unified Huntington's Disease Rating Scale Total Functional Capacity.^21^ PreHD gene carriers had a CAG repeat length ≥40 and diagnostic confidence score <4. All participants underwent the Unified Huntington's Disease Rating Scale total motor score (TMS).^21^ Disease burden score (DBS) was calculated as a measure of HD gene carriers exposure to genetic burden of disease.^22^ Healthy controls were recruited at the same time, age‐matched and gender‐matched to HD gene carriers. All participants had quantitative measures of NfL in both CSF and blood plasma and CSF *mHTT* levels and underwent an MRI scanning session.

HD‐CSF cohort participants with imaging data were included in the current study. For our analyses, we combined preHD and manifest HD gene carriers to investigate continuous change across disease progression (indexed by age × CAG interaction) rather than simple categorical prediagnosis and postdiagnosis groups, which can exclude the subtle effects of heterogeneity. Following processing and visual quality control, our final analyses included 15 control and 39 HD gene‐carrier diffusion‐weighted datasets, 15 control and 38 HD gene‐carrier myelin‐contrast images, and 18 control and 36 HD gene‐carrier quantitative T2 maps.

This study was conducted according to Declaration of Helsinki principles and was approved by the London Camberwell–St Giles Research Ethics Committee. All participants gave written informed consent prior to inclusion in the study.

### MRI Acquisition

T1‐weighted (T1W), T2‐weighted (T2W) and diffusion‐weighted MRI data were acquired on a 3T Prisma scanner (Siemens Healthcare, Erlangen, Germany) using a protocol optimized for this study. The T1W images were acquired using a 3‐dimensional Magnetization Prepared Rapid Acquisition Gradient Echo (MPRAGE) sequence with a Relaxation Time (TR) = 2000 milliseconds and Echo Time (TE) = 2.05 milliseconds; inversion time of 850 milliseconds, flip angle of 8°, matrix size 256 × 240; 256 coronal slices of 1.0 mm thickness were collected. Parallel imaging acceleration was used (GRAPPA; Generalized Autocalibrating Partial Parallel Acquisition) and 3‐dimensional distortion correction was applied to all images. T2W data for relaxometry were collected with a 3‐echo time Turbo Spin Echo sequence with TE1 = 13 milliseconds, TE2 = 81 milliseconds, TE3 = 121 milliseconds, and TR = 9400 milliseconds. Matrix size was 256 × 192 with 55 slices collected at a resolution of 1 × 1 × 2.5 mm^3^. The diffusion‐weighted MRI data were acquired using a multiband spin‐echo EPI sequence with time‐shifted RF pulses^23^ with acceleration factor 2, TR = 3600 milliseconds, TE = 65 milliseconds, flip angle = 90°, field of view = 220 mm^2^, with 72 slices collected at a resolution of 2 × 2 × 2 mm^3^. Two sets of multishell data were collected. The first consisted of no‐gradient images (b = 0) and gradient‐direction images of b = 100, b = 300, b = 700, b = 2000; the second set of data consisted of no‐gradient images (b = 0) and gradient‐direction images of b = 100, b = 500, b = 3000. A reverse phase‐encoding scan was acquired to correct for susceptibility‐induced distortion with the same acquisition parameters as the main diffusion scan.^24^

### MRI Data Processing

All scans underwent rigorous quality control, including metadata checks and visual quality control. T1W images were used for both voxel‐based morphometry (VBM) analyses and calculating myelin‐contrast maps using the MR‐Tool^25^, ^26^ in SPM12 (http://www.fil.ion.ucl.ac.uk/spm/) on a MATLAB R2012B (https://in.mathworks.com). For the VBM analyses, after N3 bias correction (SLED)^27^ each participant T1W image was segmented into different tissue classes (GM, WM, and CSF) and a group template for each created using Diffeomorphic Anatomical Registration Through Exponentiated Lie algebra (DARTEL); WM segmented images were then warped to the DARTEL template for each participant. These images were modulated to account for any volume changes that occurred during normalization and smoothed using a 4‐mm kernel with full width with half maximum. Total intracranial volume was measured using Medical Image Display and Analysis Software (MIDAS) software.^28^, ^29^ For the myelin‐contrast images, MR‐Tool uses both T1W and T2W images (here we used the T2 image acquired with TE = 13, which had the highest contrast‐to‐noise ratio).^25^, ^26^ Images were bias corrected to remove slow intensity variations and then intensity equalized using nonlinear histogram matching with external calibration from signal subtracted from nonbrain tissue. The resulting myelin‐contrast maps were then calculated as a ratio of the T1W and T2W images. For the iron‐content analyses, quantitative T2 maps were generated from the T2 scans using FMRIB Software Library (FSL),^30^ with scans for each TE aligned to produce the best image in terms of contrast to noise. A brain mask was derived from the TE = 13 scan using the Brain Extraction Tool.^31^ Segmented GM and WM images were then registered to create groupwise templates. Individual T2 maps were then registered first to their corresponding T1W image using FSL FMRIB's Linear Image Registration Tool (FLIRT) and then to the groupwise template.^32^ The resulting images were then analyzed statistically within SPM12.

Diffusion data were analyzed using the whole‐brain approach, tract‐based spatial statistics (TBSS).^33^ Prior to analysis, all diffusion datasets were checked for distortion and artefact. Susceptibility distortion was estimated using tool for estimating and correcting susceptibility induced distortions (TOPUP) in FSL and incorporated for correction within the eddy‐current distortion correction algorithm.^34^ As the data were collected using a high b‐value, multishell acquisition, we optimized the process for analysis of standard diffusion metrics by using combined eddy‐corrected lower shell data (b = 100, b = 300, b = 700) from the first set of diffusion‐weighted MRI data acquisition only. The higher shell value of b = 2000 was excluded as the diffusion signal at this b‐value is no longer suitable for modeling diffusion tensors.^35^ The tensor was fit to the combined shell data using fits a diffusion tensor model at each voxel (DTIFIT) and the output FA, MD, RD, and AD maps were included in the TBSS analysis. For the analysis of NODDI metrics, diffusion data from both acquisitions were combined using all shells.^7^ The NODDI model fitting was then applied to the eddy‐corrected data for all shells and neurite (axonal) density index (NDI) and orientation dispersion index (ODI) maps included in the TBSS analysis.

### Sample Collection, Processing, and Biofluid Marker Quantification

Sample collections were standardized to time of day, 12‐hour fast, plasticeware and processed within 15 minutes from collection as previously described.^15^, ^18^ All samples were analyzed blinded to disease status. *mHTT* levels in CSF were measured in triplicate using a single‐molecule counting immunoassay (Singulex, CA) as previously described.^18^ As expected, *mHTT* could not be detected in control participants. CSF levels of NfL were quantified in duplicate using the neurofilament (NF)‐ light enzyme‐linked immunosorbent assay (ELISA) according to the manufacturer's instructions (UmanDiagnostics, Umeå, Sweden). Plasma NfL concentration was measured in duplicate with ultrasensitive single‐molecule array (Simoa) technology—the commercially available NF light kits, as per the manufacturer's instructions (Quanterix, Lexington, MA).

### Statistical Analyses

Demographic and clinical information were analysed using independent *t* tests and chi‐squared tests where appropriate in SPSS version 22 (IBM Corp., Armonk, NY). For VBM analyses, group comparisons were performed for WM volume between control and HD gene carriers using linear regression models within SPM12. Regression analyses were then performed correlating WM volume with NfL plasma, NfL CSF, and *mHTT* levels independently in the HD‐only group. Similarly, group analysis was performed in SPM12 to compare T1:T2 weighted (myelin contrast) and quantitative T2 maps between control and HD gene carriers. Regression analyses were then performed in the HD‐only group to investigate correlations between T1:T2 weighted (myelin contrast) and NfL plasma, NfL CSF, and huntingtin *mHTT* levels independently; these analyses were then repeated for the quantitative T2 maps in SPM12. All analyses are presented at the multiple comparison corrected level at *P* < 0.05 (family‐wise error [FWE]). Following TBSS processing, diffusion maps for each metric were analyzed by means of permutation tests using FSL Randomise. Similarly to the previous analyses, group comparisons were performed between control and HD gene carriers for each diffusion metric (FA, MD, AD, RD, NDI, ODI), and then correlations between each diffusion metric and NfL plasma, NfL CSF, and *mHTT* levels were performed independently. All TBSS results are presented at *P* < 0.05 Threshold Free Cluster Enhancement (TFCE) cluster corrected unless stated.^36^ For all analyses, age and sex (and total intracranial volume for VBM) were included as covariates. All analyses were then repeated adjusting for CAG and an age–CAG interaction to test to what extent the imaging markers acted as independent predictors of NfL and mHTT levels. We applied different multiple comparison correction procedures according to the software used to perform the imaging analyses. For structural imaging analyses, SPM utilizes a standard random field theory–based correction; for diffusion imaging analyses, we applied TFCE‐cluster correction in FSL, also underscored by random field theory, but that also additionally implements cluster‐ correction. Biofluid analytes were assessed for normality and were transformed accordingly, with transformed values being used for all analyses.

## Results

For demographic and clinical information, see Table 1. Control and HD groups were matched for age and sex, but as expected differed in terms of TMS and levels of NfL (plasma and CSF) and CSF *mHTT*.

**Table 1:** Demographic and clinical information

	Controls, n = 15	HD, n = 39	Statistical Test	*P* Value
Age, y	49.46 (10.99)	51.20 (12.12)	*t* = −0.48	0.63
Sex, F (M)	7 (8)	16 (23)	chi = .14	0.77
TMS	1.67 (1.40)	23.79 (20.74)	*t* = −6.62	0.0001
DBS	N/A	355 (108)	N/A	N/A
CAG	N/A	42.54 (1.71)	N/A	N/A
*mHTT*, fM	0 (0)	65.61 (31.20)	N/A	N/A
NfL CSF, log pg/mL	6.749 (0.57)	8.120 (0.73)	*t* = −6.34	0.0001
NfL plasma, log pg/mL	2.789 (0.42)	3.865 (0.63)	*t* = −6.08	0.0001

HD, Huntington's disease; F, female; M, male; TMS, Total Motor Score; DBS, disease burden score; *mHTT*, mutant huntingtin; CSF, cerebrospinal fluid; NfL, neurofilament light; N/A, not applicable.

### Diffusion‐Based Analyses

**Figure 1:**
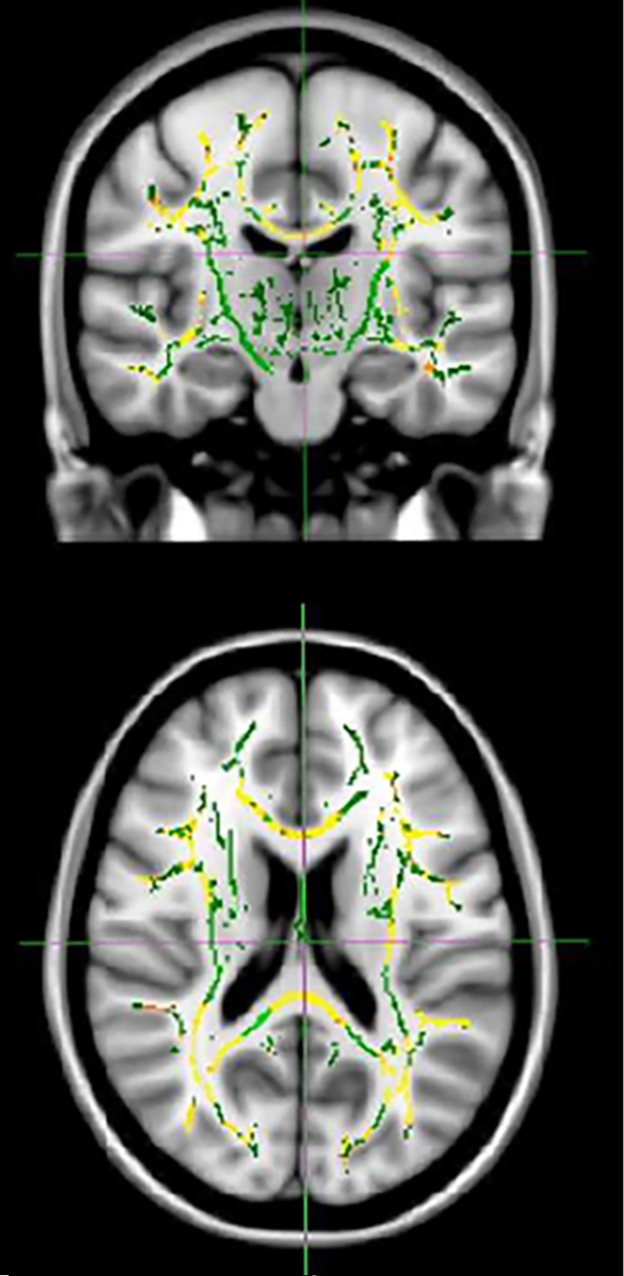
Tracts showing lower fractional anisotropy in Huntington's disease gene carriers when compared with controls. TFCE threshold of *P* < 0.05. Results (red‐yellow [lower to higher statistical values]) are projected on a white matter skeleton (green), overlaid on a customized mean fractional anisotropy image.

**Figure 2:**
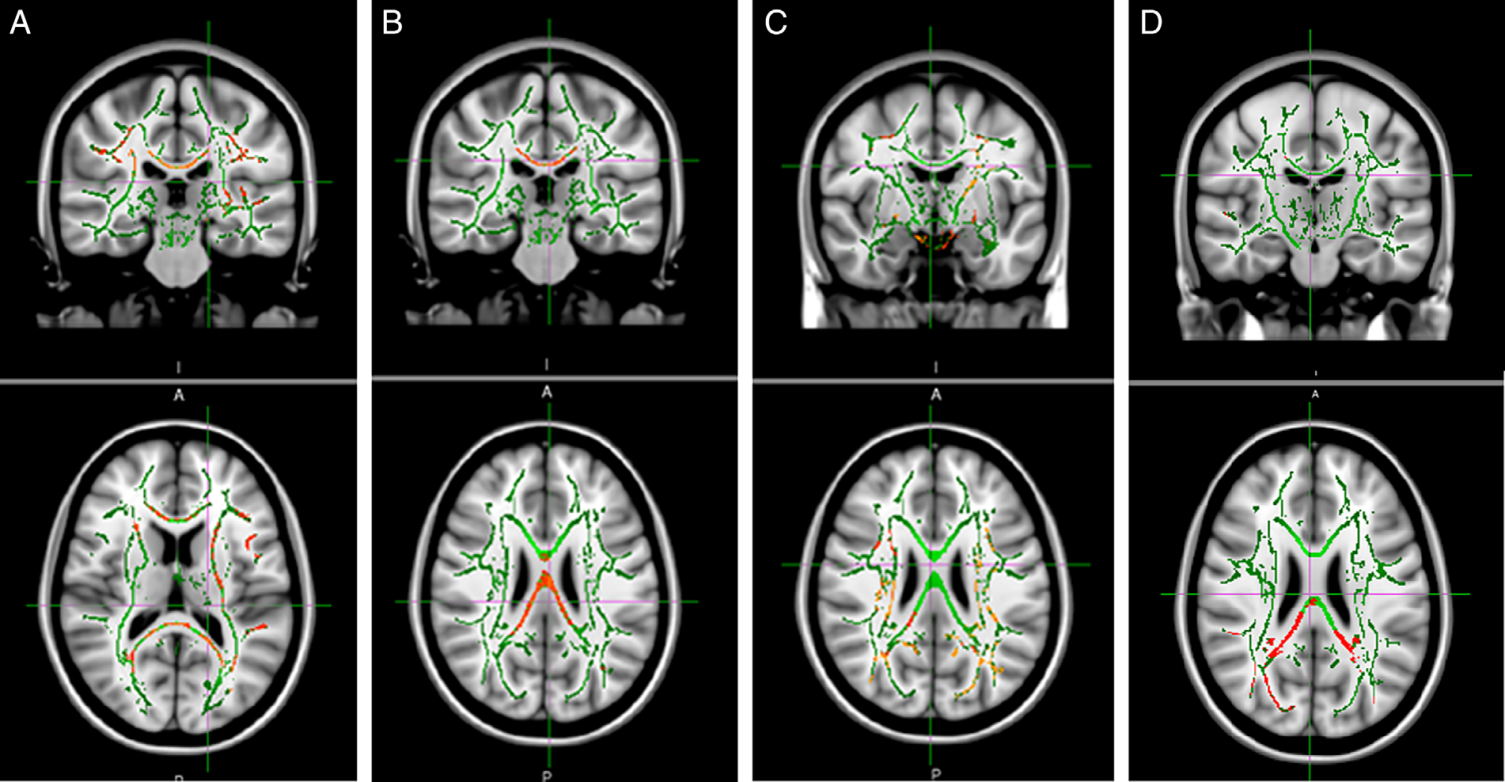
Tracts showing higher diffusivity and lower neurite density in Huntington's disease gene carriers when compared with controls. (**A**) Mean diffusivity, (**B**) radial diffusivity, (**C**) axial diffusivity, and (**D**) neurite density index TFCE threshold of *P* < 0.05. Results (red‐yellow [lower to higher statistical values]) are projected on a white matter skeleton (green), overlaid on a customized mean fractional anisotropy image.

All TBSS results are presented at *P* < 0.05 TFCE cluster corrected. FA was lower with congruent widespread higher MD, RD, and AD in the HD group when compared with controls (Figs. 1, 2A–C). There was also widespread lower NDI (Fig. 2D) in the HD group when compared to with controls. For the HD‐only group, higher FA correlated with lower DBS and TMS in the corpus callosum, mainly the splenium (and cingulum for TMS only), and there was corresponding widespread higher MD, RD, and AD across the whole brain with lower DBS and TMS. Higher ODI correlated with higher DBS across the whole brain and with higher TMS in the bilateral external capsules.

The mHTT levels correlated negatively with FA in the splenium and positively with MD in the midbody and splenium, RD in the splenium and AD in the posterior corona radiata; these findings did not survive adjustment for CAG and the age–CAG interaction. There were no statistically significant associations between any of the standard diffusion or NODDI metrics and CSF and plasma NfL levels, but there was consistent evidence of a pattern of white matter disorganization (higher diffusivity, lower FA, higher ODI) and higher NFL levels at a nonsignificant level.

### T1:T2 Weighting (Myelin‐Contrast) Analyses

All analyses are presented at the corrected level at *P* < 0.05 (FWE).The HD group showed lower myelin contrast in the left caudate when compared with controls. In the HD‐only group, lower myelin contrast correlated with lower TMS in the bilateral caudate. DBS was not significantly associated with myelin contrast. There were no significant correlations with either biofluid marker.

### Quantitative T2 Mapping

**Figure 3:**
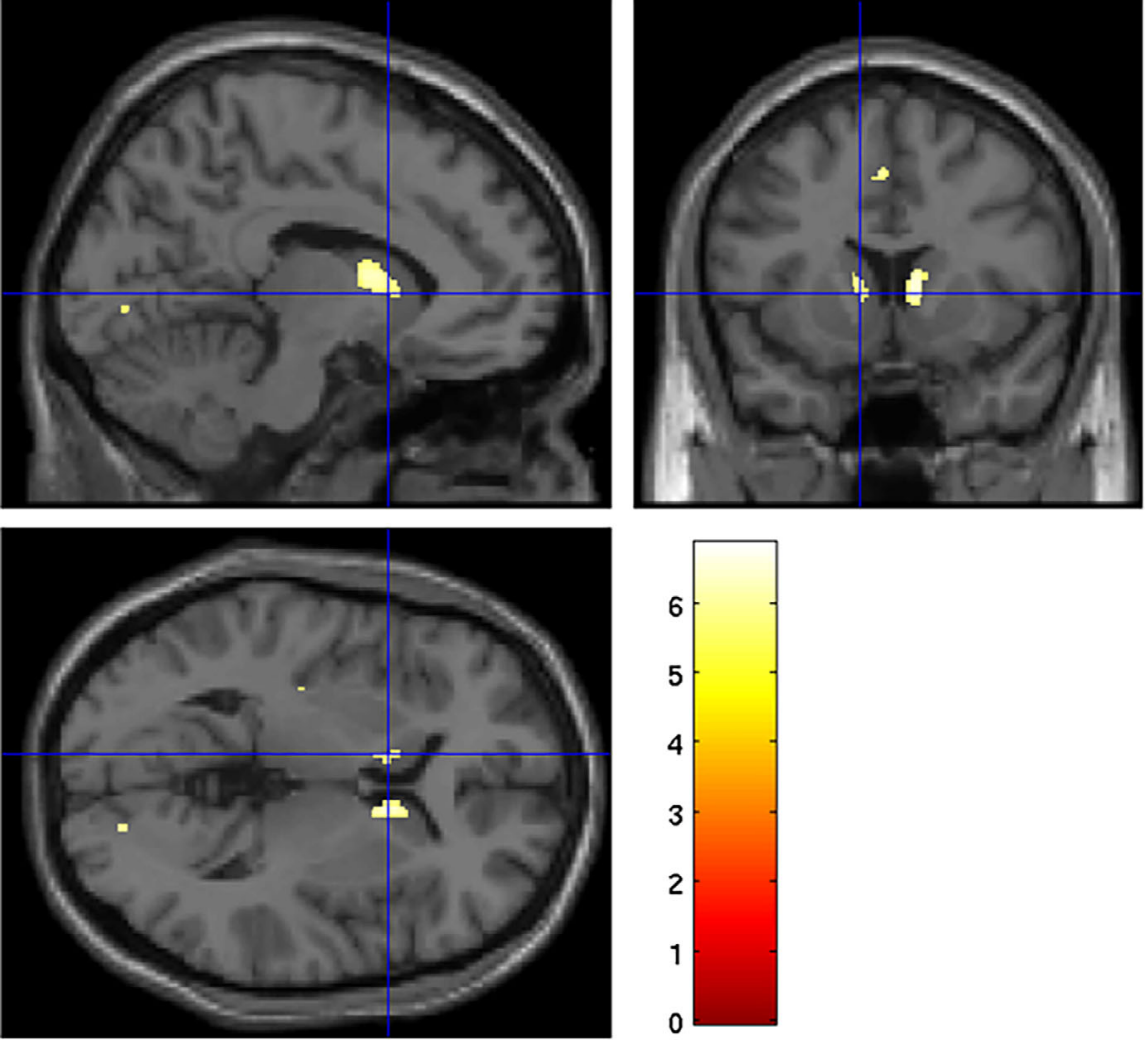
Areas where quantitative T2 is higher (lower iron content) in Huntington's disease gene carriers when compared with controls. Family‐wise error–corrected threshold of *P* < 0.05. Overlaid on a standard Montreal Neurological Institute (MNI) template.

All analyses are presented at the corrected level at *P* < 0.05 (FWE). There was higher quantitative T2 (lower iron content) in the occipital lobe, the right motor cortex, and the caudate bilaterally in the HD group when compared with controls (Fig. 3). Higher quantitative T2 (lower iron content) correlated with higher TMS in the corpus callosum and caudate bilaterally, and there were similar associations in the cerebellum with DBS. There were no significant correlations with either biofluid marker.

### VBM

All analyses are presented at the corrected level at *P* < 0.05 (FWE). WM volume was lower in premotor and motor areas in the HD group when compared with controls. In the HD‐only group, there was a negative association between bilateral WM volume around the striatum and NfL plasma levels, which survived adjustment for CAG and age–CAG interaction in the left striatum only. There was a similar negative association between left striatal WM volume and NfL CSF levels, but this did not survive adjustment for CAG and age–CAG interaction.

## Discussion

Given the role of WM degeneration in HD pathology, we have used both neuroimaging and biofluid markers to provide a more complete characterization of WM microstructure during the course of HD. We examined MRI‐based changes in diffusivity as a measure of WM connectivity using both standard DTI modeling and a novel diffusion model designed to interrogate more explicity the biological properties underlying WM differences, plus proxy measures of myelin and iron content. We then looked at the relationships between these imaging metrics and biofluid markers of neuronal damage and genetic–pathological burden, namely, NfL (sampled from both blood plasma and CSF) and CSF concentrations of mHTT, the pathogenic agent in HD. We identified widespread higher diffusivity levels coupled with lower FA and neurite density in HD gene carriers when compared with controls. Furthermore, lower FA and higher diffusivity were strongly associated with clinical measures of HD progression, while lower FA was also associated with higher mHTT levels. Myelin contrast and quantitative T2 were higher (lower iron content) in the HD group when compared with controls and similarly both highly correlated with clinical burden. There were, however, no significant associations of myelin contrast or quantitative T2 with either biofluid marker.

HD has largely been characterized by macrostructural changes, with neuronal loss occurring in the striatum and WM during premanifest stages, eventually spreading to the cortex. Despite extensive investigation of WM microstructure, the underlying biological bases of these macrostructural changes are still relatively unknown. By using multimodal imaging to investigate not only connectivity indexed by diffusion but also proxy measures of myelin contrast and iron content, in conjunction with biofluid markers of axonal degeneration, we can begin to formulate a more complete picture of the effects of HD pathology on the living human brain.

Robust evidence across a number of HD cohorts has shown WM disorganization during the earliest disease stages.^37^, ^38^, ^39^ Here, we have replicated this using whole‐brain voxelwise analysis, showing higher levels of diffusivity in HD gene carriers both when compared with controls and when correlated with clinical markers of disease severity, that is, DBS and TMS. However, standard diffusion metrics, such as FA, are nonspecific in terms of the biological properties underlying WM microstructure. In the current study, therefore, we have used a novel analysis technique, NODDI, which models intraneurite and extraneurite spaces to determine more precise biological changes in terms of axonal density and dispersion. As expected, NODDI diffusion metrics were sensitive to HD pathology. We found that axonal density (NDI) was lower in HD patients when compared with controls, similar to that seen in preHD,^5^ indicating that axonal degeneration may underlie the changes that occur in WM organization within HD. Furthermore, we found that in HD axonal dispersion (ODI) was higher in the external capsule, the white matter tract surrounding the striatum at a lower level of statistical significance (*P* = 0.06, FWE corrected), which correlated with clinical progression at a similarly lower level of significance (*P* = 0.06, FWE corrected), indicating that axonal proliferation in striatal white matter tracts may be related to disease stage. Given that axonal dispersion in WM tracts surrounding the basal ganglia has been shown to be lower in preHD,^5^ it would be interesting to investigate further when and to what extent fibers become dispersed during HD.

While there is evidence for axon‐driven WM degeneration, in particular lower axonal density, we wanted to characterize these changes more fully by examining the relationship between this diffusivity‐based evidence of WM disorganization and NfL, a biofluid marker of axonal degeneration that is highly correlated with HD progression.^15^ We did not identify any significant relationships between any of the structural or diffusion metrics and NfL. However, there was a clear pattern of increased NfL and increased white matter disorganization. We saw a negative relationship between FA and NfL (CSF) levels in the corpus callosum at a significance level of *P* = 0.06, FWE corrected—interesting given the evidence of early callosal degeneration in HD.^40^, ^41^ This was coupled with a pattern of higher NfL levels and both higher diffusvity, that is, MD, AD, and RD and increased axonal dispersion in the external capsules surrounding the striatum. Together these findings indicate that white matter disorganization in HD may be associated with axonal degeneration. However, we proceed with caution given that these findings were only evident below our specified statistical threshold and therefore, this hypothesis requires requires further testing in a similarly well‐characterized but larger HD cohort, potentially over time.

Mutant HTT was also highly and robustly correlated with increased MD, AD, and RD, and although the specific nature of mHTT effects on WM microstructure is unclear, it would appear that higher levels of mHTT reflect some degree of WM degeneration. Our findings are of particular interest given that WM change in HD seems to occur considerably earlier than that of cortical matter atrophy and supports a hypothesis of axonal degeneration in HD. Interestingly, none of our correlations survived correction for the age × CAG interaction, signifying that axonal degeneration is unlikely to be independent of disease progression but, rather, is associated with the worsening effects of pathology.

Myelin contrast (indexed by the T1:T2 weighting maps) and quantitative T2 changes in our HD cohort were less widespread than those associated with diffusivity. There was evidence of lower myelin contrast in the caudate and higher quantitative T2 (indicative of lower iron content) in the caudate and select cortical regions in the combined HD group when compared with controls. Furthermore, there was also lower myelin contrast and higher quantitative T2 (indicative of lower iron content) in the bilateral caudate as motor performance deteriorated in the HD‐only group. This combination of potentially lower iron content and myelin contrast suggests a reduction in the presence of iron‐based oligodendrocytes, which may indicate demyelination in striatal regions. Given the evidence for axonal degeneration, however, it would appear that demyelination is less associated with HD pathology than axonal breakdown, particularly given the lack of correlation between both iron and myelin and NfL or mHTT. However, we do acknowledge that both the myelin‐contrast ratio and quantitative T2 mapping are proxy measures of myelin and iron content, respectively. Quantitative T2 mapping, for example, may not be adequately sensitive to detect changes driven by changes in NfL levels, explaining the lack of association. Alternative MRI techniques, such as multiparameter mapping^42^ and quantitative susceptibility mapping,^43^ may offer more sensitive and/or biologically specific approaches to further investigate the role of demyelination and iron toxicity in HD pathology.

Plasma NfL increases appeared to show a greater association with increased diffusivity measures than CSF NfL. However, these patterns were only visible at a lower corrected threshold and require further investigation in a larger HD cohort.

Using a HD cohort of both premanifest and manifest gene carriers, we have shown that WM diffusivity is higher in HD than control participants, correlating clearly with measures of disease burden, including clinical scores, such as DBS and TMS, and the toxic source of disease, mHTT itself. Proxy measures showed lower myelin contrast and iron content in the HD group when compared with controls, suggesting potential demyelination within striatal regions. By investigating white matter microstructure, underlying biological properties that may drive microstructural change, such as myelin and iron, and products of WM damage including NfL, we have presented a detailed characterization of WM change in HD.

## Author Roles

(1) Research Project: A. Conception, B. Organization, C. Execution; (2) Statistical Analysis: A. Design, B. Execution, C. Review and Critique; (3) Manuscript Preparation: A. Writing of the First Draft, B. Review and Critique.

S.G.: 1C, 2A, 2B, 2C, 3A, 3B

E.B.J.: 1C, 2B, 3B

L.M.B.: 1B, 1C, 3B

F.B.R.: 1B, 1C, 3B

A.H.: 2B, 3B

J.M.: 2B, 3B

D.T.: 1B, 3B

H.Z.: 1B, 3B

E.d.V.: 1B, 1C, 3B

S.J.T.: 3B

G.R.: 3B

R.I.S.: 2B, 2C, 3B

E.J.W.: 1A, 1B, 2C, 3B

## Disclosures

### Ethical Compliance Statement

This study was conducted according to Declaration of Helsinki principles and was approved by the London Camberwell–St Giles Research Ethics Committee. All participants gave written informed consent prior to inclusion in the study. We confirm that we have read the Journal's position on issues involved in ethical publication and affirm that this work is consistent with those guidelines.

**Funding Sources and Conflicts of Interest:** This work was primarily supported by the Medical Research Council (MR/M008592/1). S.G., E.B.J., R.I.S., G.R., and S.J.T. receive support from a Wellcome Trust Collaborative Award (200181/Z/15/Z). F.B.R. is supported by Cure Huntington's Disease Initiative (CHDI) Foundation. E.D.V. is supported by the Wellcome Engineering and Physical Sciences Research Council Centre for Medical Engineering (WT 203148/Z/16/Z).

**Financial Disclosures for the Past 12 Months:** Via University College London (UCL) Consultants, a wholly‐owned subsidiary of UCL, E.J.W. has participated in consultancies and scientific advisory boards with Hoffmann‐La Roche Ltd, Ionis Pharmaceuticals, Shire, Novartis, Mitoconix, PTC Therapeutics, and Wave Life Sciences and is an investigator on the IONIS‐HTTRx (RG6042) program. His host clinical institution, UCL Hospitals NHS Foundation Trust, has received funds as compensation for conducting clinical trials for Ionis Pharmaceuticals, Pfizer, and Teva Pharmaceuticals.
